# *Trichuris muris* and comorbidities – within a mouse model context

**DOI:** 10.1017/S0031182021000883

**Published:** 2021-12

**Authors:** Kelly S. Hayes, Richard K. Grencis

**Affiliations:** Lydia Becker Institute of Immunology and Inflammation, Wellcome Trust Centre for Cell Matrix Research and Faculty of Biology, Medicine and Health, University of Manchester, Manchester, UK

**Keywords:** cancer, co-infections, comorbidity, *Trichuris muris*

## Abstract

*Trichuris muris* is a mouse intestinal parasitic nematode that inhabits the large intestine of its host and induces a strong immune response. The effects of this strong anti-parasite response can be found locally within the intestinal niche and also systemically, having effects on multiple organs. Additionally, the anti-parasite response can have multiple effects on infectious organisms and on microbiota that the host is harbouring. It has been shown that Th1 responses induced by *T. muris* can affect progression of bowel inflammation, cause colitic-like intestinal inflammation, reduce barrier function and intestinal mucosal responses. In the brain, *T. muris* can exacerbate stroke outcome and other neurological conditions. In the lung, *T. muris* can suppress airway inflammation and alter immune responses to other parasites. Additionally, *T. muris* induced responses can inhibit anti-tumour immunity. Although this parasite maintains a localized niche in the large intestine, its effects can be far-reaching and substantially impact other infections through modulation of bystander immune responses.

## Graphical abstract

**Figure:**
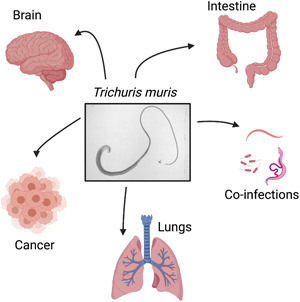

## Introduction

*Trichuris muris* is a mouse intestinal parasitic nematode used as an experimental model for the human counterpart, *T. trichiura*. This nematode is one of the four major soil-transmitted helminths that infect 1.5 billion people worldwide causing significant morbidity (WHO, 2020). These diseases bear a huge impact on the quality of life of infected people and on the economic growth of infected communities (Hotez *et al*., 2014).

**Fig. 1.**
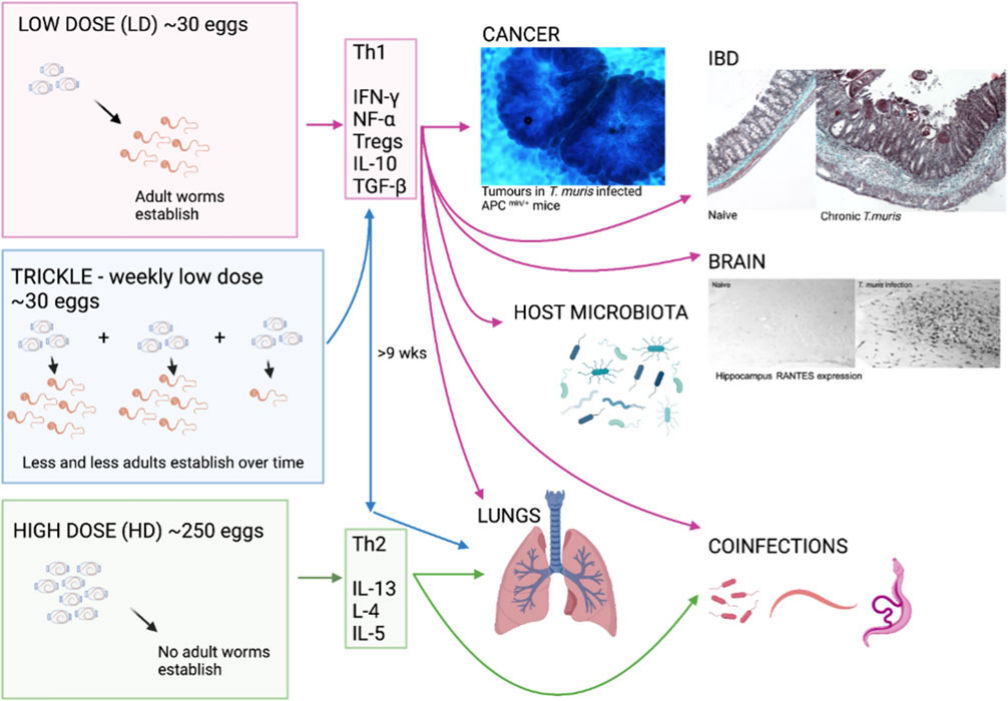
The whipworm *T. muris*, though caecal dwelling, can affect many other systems in the body. The immune response to *T. muris* is dose-dependent with different cytokines being produced in response to the different doses of eggs given which can lead to chronic infection (Th1) or expulsion (Th2). Each of the immune responses to the differing doses of eggs can impact different systems in the body as depicted by the arrows. As pictured, tumours are increased in size and number in a cancer model with chronic *T. muris*, pathology is increased in chronic infection and shows similarity to IBD, and hippocampus RANTES expression is increased with chronic *T. muris* infection. Changes in microbiota, lung effects and effects on other infections are also apparent with *T. muris* infection. (Created with BioRender.com)

*T. muris* inhabits the large intestine and caecum of the host, with adult parasites living with their anterior half tunnelled into the host epithelium and their posterior free in the lumen to facilitate egg deposition (Cliffe and Grencis, 2004). The immune response to *T. muris* in mice is very well characterized and there is a distinct polarization of immune response in resistant and susceptible strains of mouse (Else and Grencis, 1991; Else *et al*., 1992). Resistant animals produce high levels of interleukin 13 (IL-13) and associated T helper type 2 (Th2) cytokines in response to infection (Fig. 1), which are essential for parasite expulsion *via* mechanisms such as epithelial cell turnover and mucin production and muscle contraction (Khan *et al*., 2003; Cliffe *et al*., 2005; Hasnain *et al*., 2010; Chen *et al*., 2021). In contrast, a susceptible animal produces high amounts of interferon-*γ* (IFN-*γ*) and Th1 associated cytokines (Fig. 1) that leads to chronic infection, enabling the parasite to establish to maturity within the large intestine and release eggs into the environment, thereby perpetuating infection. Trickle infections can also be used to more closely mimic a natural infection of repeated low-dose exposures. Weekly trickle infections promote an initial Th1 response but this changes to a dominant Th2 response after 9 weeks (Fig. 1), which prevents any further establishment of worms (Glover *et al*., 2019). Chronic infection, either in genetically susceptible mice or due to a low-dose infection and its associated Th1 response, are associated with dysregulation within the gut, such as crypt hyperplasia and apoptosis (Cliffe *et al*., 2007) together with a regulatory response that is required to limit worm-driven pathology (D'Elia *et al*., 2009; Grencis *et al*., 2014; Duque-Correa *et al*., 2019). Interestingly, reducing T regulatory (Treg) cells early on during a low-dose infection does have a small but significant effect on the capacity to expel parasites and subsequently intestinal pathology is reduced, suggesting that this induced Treg response is of benefit to both the host and to the parasite (Sawant *et al*., 2014). However, this effect on parasite expulsion was lost if Tregs were depleted once infection had become established (Sawant *et al*., 2014). A key cytokine produced by CD4^+^ T cells IL-10, is critical in host survival during *T. muris* infection (Schopf *et al*., 2002) although whether Tregs are the major source of IL-10 during *T. muris* infection is unclear. TGF-*β* is another regulatory cytokine that is produced during *T. muris* infection that can dampen CD4^+^ T cell responses (Li and Flavell, 2008). As with the effects of an early reduction in Tregs, early ablation of TGF-*β* during a low-dose infection again caused a significant, although partial, reduction in worm numbers (Worthington *et al*., 2013). When the ability of dendritic cells to induce TGF-*β* was prevented, mice were able to clear a low-dose infection efficiently although this did not seem to be dependent upon the generation of Tregs (Worthington *et al*., 2013). Thus, it appears that the regulatory response generated by *T. muris* is complex and involves CD4^+^ T cells, Tregs, IL-10 and TGF-*β* contributing to the net result of a chronic infection with controlled intestinal inflammation. This review will discuss the differing effects that either low-dose or high-dose intestinal *T. muris* infection can have on both enteral and systemic responses in the host (Fig. 1).

## Intestinal inflammation

Inflammatory bowel disease (IBD) in humans represents broadly two distinct immunological conditions; Crohn's disease and ulcerative colitis. Disease onset is prompted in genetically susceptible individuals by atypical responses to microbiota or environmental cues such as diet and stress (Guan, 2019). The influence of human trichuriasis upon IBD has received little attention, with notable exceptions (Broadhurst *et al*., 2010). This study followed pathological and immunological changes in an individual with ulcerative colitis prior to and following self-treatment with *T. trichiura*. The data supported a modulatory role for whipworm infection upon disease severity with infection associated with disease remission. Due to the intestinal niche that *Trichuris* species inhabit, an effect upon inflammatory disease of the large intestine in the host might be expected. Mechanistically this can be explored in the mouse using *T. muris* together with murine models of IBD. It is plausible that *T. muris* infection may cause IBD symptoms while the host immune response to the parasitic infection could have implications on progression of intestinal inflammation. Specifically, it is known that a low-dose infection of ~20 *T. muris* eggs will proceed to chronicity (Fig. 1), even in normally resistant strains of mouse, leading to an IFN-*γ*/Th17-driven disease (Levison *et al*., 2010) that is controlled by a concomitant IL-10 response (Grencis *et al*., 2014). Indeed, IL-10 knock-out (KO) and IL-10R KO mice develop severe pathology in response to *T. muris* infection (Schopf *et al*., 2002; Duque-Correa *et al*., 2019). This low-dose infection regime can be used to mimic colitis, leading to both phenotypic and transcriptional similarities to other widely used models of IBD (Levison *et al*., 2010; Foth *et al*., 2014). Of 32 genes that are known to be transcriptionally different during IBD, 30 are also found to be upregulated in the CD4^+^CD45RB T cell transfer model of colitis (te Velde *et al*., 2007). Nineteen of these 30 genes, including IFN-*γ*, were also found to be upregulated in chronic *T. muris* infection (Levison *et al*., 2010). Indeed, chronic *T. muris* infection shows a degree of similarity to all mouse models of Th1-driven colitis, both phenotypically and transcriptionally, though the degree of similarity does vary from model to model (Levison *et al*., 2010). Additionally, it has been shown that *T. muris* pathology and Crohn's disease have overlapping QTL regions – overlapping regions of DNA suggesting common genetic parameters (Levison *et al*., 2013). To exemplify this, the role of two different cytokines have been shown to be important in both *T. muris* and colitis, IL-27 and IL-13. IL-27 is a potent stimulator of Th1 responses (Pflanz *et al*., 2002) and is more highly expressed in patients with IBD (Nemeth *et al*., 2017). However, IL-27 is also known to regulate Th17 responses and to stimulate IL-10 production and Treg generation (Awasthi *et al*., 2007; Yoshida and Hunter, 2015). Oral delivery of IL-27 recombinant bacteria can ameliorate T cell transfer-induced colitis in mice (Hanson *et al*., 2014) whilst a *T. muris* infection in an IL-10/IL-27 KO mouse leads to less severe pathology than seen in the IL-10 KO control due to a decreased pro-inflammatory profile (Villarino *et al*., 2008). Additionally, WSX-1-deficient animals, that lack the functional receptor for IL-27, mount a heightened Th2 response to infection and show an accelerated expulsion of the parasite (Artis *et al*., 2004; Bancroft *et al*., 2004). Despite these contrasting results, known IL-27 gene polymorphisms in IBD patients (Li *et al*., 2009; Wang *et al*., 2014) make this cytokine an intriguing IBD therapy candidate (Andrews *et al*., 2016). In contrast, IL-13 is a Th2/Type 2 cytokine (Minty *et al*., 1993) that is upregulated during an acute resolving *T. muris* infection (Bancroft *et al*., 1998). IL-13 is a potent suppressor of Th1 responses in humans (de Waal Malefyt *et al*., 1993; Wynn, 2015), although its role in IBD is complex. Crohn's disease is principally a Th1 and IFN-*γ* driven condition whilst ulcerative colitis is associated with increased Th2 cytokines such as IL-5 and IL-13 (Fuss *et al*., 1996, 2004). *T. muris* infection in IL-10/IL-13R*α*2 KO mice has been used to highlight the importance of IL-13 in controlling *T. muris*-induced pathology. IL-13R*α*2 is the decoy receptor for IL-13 and reduces the bio-availability of IL-13 (Mentink-Kane and Wynn, 2004). When infected with *T. muris*, IL-10/IL-13R*α*2 KO mice have a decreased morbidity and mortality as compared to IL10 KO mice (Wilson *et al*., 2011) demonstrating the protective role of IL-13. In support of this, recent studies have shown that IL-13 acts to mediate recovery and repair in the gut following dextran sulphate sodium (DSS)-induced colitis, which is Th1 driven, as disease was improved in both IL-13R*α*2 KO mice and in mice treated with a neutralizing IL-13R*α*2 antibody (Karmele *et al*., 2019). Additionally, transcripts for IL-13R*α*2 have been found to be elevated in human IBD biopsies suggesting a protective role for IL-13 in these patients (Arijs *et al*., 2009, 2010). Similarly, patients expressing a more active variant of IL-13, with a reduced affinity to the IL-13*α*2 decoy receptor, had a lower risk of developing Crohn's disease (Karmele *et al*., 2019).

Although *T. muris* infection can cause varied components of intestinal inflammation, the Treg response (D'Elia *et al*., 2009; Worthington *et al*., 2013; Sawant *et al*., 2014; Duque-Correa *et al*., 2019) that it also initiates has been taken as a basis for a potential approach to treat IBD. The pig whipworm *T. suis* has been used in human trials for treatment of both Crohn's disease and ulcerative colitis with resulting remission of disease in some patients in small cohort studies (Summers *et al*., 2005*a*, 2005*b*) although no clinical improvement was seen in a larger cohort study (Schölmerich *et al*., 2017). Although the exact mechanisms of action are unknown, excretory/secretory (E/S) products of *T. suis* on epithelial cells *in vitro* have been shown to elicit IL-6 and IL-10 secretion (Parthasarathy and Mansfield, 2005). Additionally, when *T. suis* E/S products were added to bone-marrow-derived macrophages and dendritic cells, there was a reduction in secretion of pro-inflammatory cytokines and a strong enhancement of IL-10 secretion (Leroux *et al*., 2018). Remission of ulcerative colitis, following self-infection with *T*. *trichiura*, was associated with a marked elevation in IL-22 (an IL-10 family member) producing T cells which were hypothesized to promote intestinal repair by increasing goblet cell numbers and mucus production (Broadhurst *et al*., 2010).

### Barrier function in the intestine

During infection, *T. muris* is known to cause epithelial dysregulation in the large intestine (Artis *et al*., 1999; Cliffe *et al*., 2007), a process which is also observed in human IBD (Strober *et al*., 2007). *T. muris* induced TNF-*α* and IFN-*γ* production drive apoptosis within the caecal crypts of the large intestine (Artis *et al*., 1999), which is thought to be in response to IFN-*γ*-induced epithelial cell hyperproliferation that also occurs (Cliffe *et al*., 2007) thus leading to a perturbation in intestinal homeostasis. Infection with *T. trichiura*, the human whipworm, may cause trichuris dysentery syndrome (Cooper *et al*., 1990) in children, which is also associated with an increase in TNF-*α* production by mucosal macrophages (MacDonald *et al*., 1994). Increased intestinal apoptosis is also known to lead to a dysregulation of barrier integrity with an associated increase in epithelial permeability in IBD patients (Schulzke *et al*., 2006; Mankertz and Schulzke, 2007). During acute *T. muris* infection (whereby the worms are expelled before chronicity, Fig. 1), there is an accumulation of epithelial mast cells in the large intestine (Sorobetea *et al*., 2017). Mast cells produce mast cell protease-1 (MCPt-1) (Metcalfe *et al*., 1997) and indeed, acute *T. muris* infection is associated with an increase in MCPt-1 both systemically and locally in the large intestine, which is associated with a loss of barrier integrity leading to increased epithelial permeability (Sorobetea *et al*., 2017). *T. muris* infection in IL-10 KO mice is known to result in marked mortality and morbidity including a loss of Paneth cells and an absence of mucus (Schopf *et al*., 2002). Pathology in IL-10 KO and IL-10/IL-4 KO mice is also associated with bacterial outgrowth as broad-spectrum antibiotic treatment enhances survival (Schopf *et al*., 2002). Duque-Correa *et al*. (2019) also showed that IL-10 signalling had a protective effect on loss of barrier integrity leading to bacterial translocation. It is also known that *T. suis* E/S can affect barrier integrity by reducing the expression of tight junction proteins (Hiemstra *et al*., 2014) although whether this is also a function of *T. muris* E/S is unknown. However, Hasnain *et al*. (2012) showed that adult *T. muris* E/S was able to degrade intestinal mucins and *T. muris*-induced changes in the intestinal mucus barrier have also been demonstrated that may act to increase intestinal permeability (Hasnain *et al*., 2010, 2011). Infection itself can lead to thickening of the glycocalyx, the glycoprotein and glycolipid covering of the intestinal epithelial cells (Linden *et al*., 2008) likely due to the increased production of mucin proteins. However, there is also a decreased glycoprotein content within the mucosal barrier during chronic infection that may allow increased contact of the intestinal microbiota with intestinal epithelial cells (Hasnain *et al*., 2011). Congruous to this, chronic *T. muris* infection can also alter the host intestinal microbiota (Holm *et al*., 2015; Houlden *et al*., 2015) and it is known that a modification in the composition and function of the gut microbiota can also change intestinal permeability (Gomaa, 2020).

### Microbiota changes in the intestine

Changes in microbiota during a *T. muris* infection are evident from as early as only day 14 post-infection (p.i.). By the time that infection has reached patency (more than day 33 p.i.), there are significant changes in the composition and diversity of the microbiota (Fig. 1) (Holm *et al*., 2015; Houlden *et al*., 2015). There was a general shift in the microbiota to a decreased number of bacteria in the Bacteroidetes phyla and an increased number of Gram-positive Lactobacillaceae. Such changes in the microbiota appear to be of benefit to the parasite and changes were transitory and required the presence of the parasite to be maintained (White *et al*., 2018). In contrast, changes in microbiota composition in an outbred strain of mouse with a chronic *T. muris* infection led to an increase in bacterial invasion of the host intestinal epithelium (Schachter *et al*., 2020). Interestingly, infection-induced microbiota changes can also promote resistance to damage. In a colitis-susceptible strain of mouse (NOD2 KO), it has been established that overgrowth of *Bacteroides vulgatus* leads to intestinal abnormalities (Ramanan *et al*., 2014). However, acute infection with *T. muris*, that drives a Th2 response and a mucus response, led to an increase in *Clostridia* strains of bacteria that inhibited *B. vulgatus* colonization and the resulting *B. vulgatus*-driven abnormalities (Ramanan *et al*., 2016). The microbiota of the host can also directly influence pathogenesis of *T. muris* as antibiotic treatment of chronically infected IL-10 KO animals, although experiencing similar pathology to control animals, had a significantly reduced mortality (Kopper *et al*., 2015). Chronic infection induced changes to microflora have also been shown in *T. suis* infected pigs (Li *et al*., 2012) although there is contrasting evidence as to whether the human whipworm also drives microflora changes (Cooper *et al*., 2013; Ramanan *et al*., 2016).

### *Trichuris* effects distal to the site of infection

Despite its intestinal epithelial location, the effects of *T. muris* infection are not only restricted to the site of infection. Chronic *T. muris* infection can modulate responses to chemical skin sensitizers applied to the ear of the mouse. Suppression of local cellular/cytokine Th1/pro-inflammatory responses and ear pathology were observed when using a Th1-promoting compound [2,4-dinitrochlorobenzene (DNCB)] although no depression in IL-13, or ear swelling was noted after sensitizing with the Th2-promoting compound trimellitic anhydride (TMA). Interestingly, the suppression of pathology after DNCB treatment was associated with a reduction in egress of dendritic cells (DCs) from the skin coincident with elevated IL-10 production and a slight increase in CD4^+^FoxP3^+^ cells in the draining lymph node (Grencis *et al*., 2014). Movement of DCs from the skin to the draining lymph node has been shown to be dependent on local proinflammatory cytokines which can be inhibited by IL-10 production (Cumberbatch *et al*., 2000).

### *T. muris* effects in the lungs

Chronic *T. muris* infection which drives a strong Th1 response in the intestine, has also been shown to drive the production of IFN-*γ* (by Th1 cells) and IL-10 (myeloid cells) in the lung of the host (Fig. 1), and so has the potential to suppress the development of Type-2-driven airway inflammation (Chenery *et al*., 2016). The increased Th1 type response in the lung was able to reduce the lung response to both papain and house-dust mite, together with a reduced eosinophil infiltration and reduced lung mucus production. IL-17 is another cytokine known to be increased in complex asthma and may contribute to disease progression (Doe *et al*., 2010): additionally, IL-17 is critical for neutrophil expansion and remodelling of lung tissue and may contribute to disease progression in other chronic respiratory conditions (Gurczynski and Moore, 2018). A high-dose infection of *T. muris*, that induces a Th2 response (Fig. 1), can promote a mixed IL-17 and Th2-type immunity to the parasite (Wilson *et al*., 2011). Induction of Th2 cytokines can also be seen in the host lung following infection with a high dose of *T. muris*, however, this is dependent on IL-17 production and is ablated in an IL-17 KO animal (Ajendra *et al*., 2020). Interestingly, this IL-17-dependent suppression of IFN-*γ*, which allowed the promotion of type-2 immune responses, was only apparent in the host lung and was not seen in the intestine. Additionally, a secreted product from *T. muris*, p43, is able to bind to IL-13 *in vitro* and *in vivo* (Bancroft *et al*., 2019). When given to mice intranasally with IL-13, p43 reduced the percentage of RELM-*β* positive interstitial lung macrophages as compared to mice treated with IL-13 only. The effects of p43 are further reviewed in this special issue by Bancroft & Grencis. By-stander effects of *Trichuris* infection in the lung are also seen with other species of *Trichuris*. *T. suis* ova treatment in a grass-pollen allergy clinical trial increased Th2 and IL-10 production in patients although this did not affect allergen-specific cytokine responses (Bourke *et al*., 2012). Interestingly, treatment of ovalbumin-sensitized mice with *T. suis* larval E/S proteins suppressed airway hyperreactivity and bronchiolar inflammation, partially mediated by E/S-induced IL-10 secretion (Ebner *et al*., 2014). Whether *T. trichiura* has similar abilities to modulate inflammation is uncertain and there are conflicting results in the literature (Rodrigues *et al*., 2008; Alcântara-Neves *et al*., 2010; Gonçales *et al*., 2020).

### *T. muris* cerebrovascular and neurodegenerative disease

It is well established that infection and systemic inflammation are risk factors for ischaemic brain damage (stroke) and can also affect the progression of some neurodegenerative disorders (He *et al*., 2020).

Using transient middle cerebral artery occlusion as a model of stroke it was shown that a chronic low-dose *T. muris* infection, which drives a Th1 response (Fig. 1), dramatically exacerbated brain damage caused by experimental stroke (Dénes *et al*., 2010). Infection led to an increase in pro-inflammatory mediators in the brain and surrounding tissue together with an altered Treg response. Infected mice had elevated Th1-associated cytokines and chemokines after cerebral artery occlusion however, only CCL5 (RANTES) stayed significantly increased after 48 hours post-stroke. Anti-RANTES treatment prevented the infection-driven exacerbation of stroke-induced damage. Analysis of matrix metallopeptidase 9 expression in the brain showed elevated levels after stroke and infection compared to stroke alone indicating augmented vascular injury and blood−brain barrier damage in chronically infected animals. Interestingly, an acute, resolving *T. muris* infection driving a Th2 response had no effect on infarct size demonstrating that it was the Th1 milieu driven by the parasite that was detrimental rather than the parasite itself (Dénes *et al*., 2010). The detrimental effects of infection are also very much dependent on age as infarct size was found to be significantly increased in chronically infected aged mice as compared to chronically infected young mice (Dhungana *et al*., 2013). Older mice experienced an increased neutrophil recruitment and upregulation of Th1 cytokines as compared to the younger mice leading to the increased pathology seen.

As well as stroke, it has also been demonstrated that chronic *T. muris* infection can accelerate the onset of experimental clinical prion disease – a chronic, neurodegenerative disease caused by infectious proteins (Donaldson *et al*., 2020). Mice were infected with a chronic *T. muris* infection after receiving prions, timed so that the peak of parasite-driven inflammation would coincide with known pre-clinical phases of the prion infection. *T. muris* infected mice had a reduced survival time which correlated with increased pro-inflammatory cytokines in the sera and increased numbers of CD8+ cells in the brain (Donaldson *et al*., 2020). *T. muris* infection can also exacerbate neuroinflammation in models of Alzheimer's disease, a chronic neurodegenerative condition (Querfurth and LaFerla, 2010; Montacute *et al*., 2017). Infection in the Alzheimer's mouse model (3xTg-AD) led to increased levels of inflammation in the brain with increased microglia activation. Interestingly, these transgenic animals were also unable to fully expel a high-dose infection, which is normally acute and resolving (Fig. 1), together with increased Th1 cytokine levels in response to infection in the lymph node draining the large intestine (Montacute *et al*., 2017). Although not addressed in any *T. muris* infection model, *T. suis* E/S effects in experimental autoimmune encephalomyelitis, an animal model of multiple sclerosis (MS), have been assessed (Kuijk *et al*., 2012; Hansen *et al*., 2017). Intraperitoneal administration of *T. suis* E/S before disease onset significantly decreased disease severity and markedly reduced systemic Th1 and Th17 responses (Hansen *et al*., 2017). However, *T. suis* ova therapy in MS clinical trials have had mixed effects (Voldsgaard *et al*., 2015; Fleming *et al*., 2019; Yordanova *et al*., 2021).

## Trichuris and coinfections

Surprisingly little work has been carried on coinfections of *T. muris* and viral or bacterial infections though some work has been done with *Mycobacteria* and *Streptococcus*. Immunity to *Mycobacterium bovis* (*M. bovis*) infection has been shown to be negatively influenced by a *T. muris* coinfection. A high-dose *T. muris* infection, which promotes a Th2 response, down-regulated pulmonary Th1 and Treg cell responses to the bacteria (Fig. 1) (Nel *et al*., 2014) although this had no effect on bacterial proliferation and dissemination. However, *T. muris* E/S-treated human monocyte-derived macrophages prior to exposure to *M. tuberculosis* led to an M2-type polarization with reduced macrophage phagosome maturation and a resulting increased bacterial burden (Aira *et al*., 2017). In a *T. muris*-*Streptococcus pneumoniae* coinfection model, nematode infection was associated with an increased carriage of *S. pneumoniae*, though this did not reach significance, with a significant increase in dissemination of the bacteria to the lungs (Law *et al*., 2021). Anthelmintic treatment led to a smaller, though not significant, load of bacteria. This trend for a higher carriage of bacteria when coinfected with *Trichuris* was similarly seen in children harbouring *T. trichiura* (Law *et al*., 2021).

Protozoan infections such as *Plasmodium berghei*, *Trypanosoma brucei* and *Babesia microti* and *B. hylomysci* will all delay the expulsion of a high dose of *T. muris* infection, particularly at times of high parasitaemia suggesting that at least acute *T. muris* infections do not exert strong immunomodulatory effects on these co-infections (Phillips and Wakelin, 1974; Phillips *et al*., 1974).

More data are available on the effect of *T. muris* infection on other helminth infections. Experimental infection of *Nematospiroides dubius* [*Heligmosomoides polygyrus (bakerii)*], which resides in the small intestine, delayed expulsion of a high dose *T. muris* infection and enhanced survival of a trickled *T. muris* infection (Behnke *et al*., 1984). The lung, like the gut, is a mucosal surface and many helminth parasites have evolved a migratory phase through the lungs in their life cycle (Craig and Scott, 2014). Cross-talk between the lung and intestinal mucosal surfaces in terms of host immunity is particularly evident during helminth co-infections. *Nippostrongylus brasiliensis* is a rodent small intestinal dwelling parasite that migrates through the host lung before reaching maturity (Bouchery *et al*., 2017). Intestinal infection with a high dose of *T. muris*, that promotes a Th2 response and is expelled by the host (Fig. 1), reduced the number of *N. brasiliensis* larvae found in the lung at d2 post-infection (Filbey *et al*., 2019). Interestingly, mice that had been given a trickle infection of *T. muris* (initially driving a Th1 response and then a protective Th2 response) and then a *N. brasiliensis* infection, after the switch to a Th2 dominated response, had an equivalent number of larvae in the lung at d3 post-infection as WT mice (Glover *et al*., 2019). This suggests either a resolving delay in *N. brasiliensis* migration in the lung as equivalent numbers of adults were found in the intestine (Glover *et al*., 2019) or a qualitative difference in the Th2 response initiated by a high dose as compared to a trickle infection.

*T. muris*-induced alteration in the lung cytokine expression has also been demonstrated in co-infection with *Schistosoma mansoni* (Bickle *et al*., 2008). *S. mansoni* is a trematode that causes chronic infection in mice, causing pathology in the lungs as it migrates (Boros, 1989). Chronic infection with *T. muris* led to a reduced trapping of larvae during their skin-to-lung migration associated with an altered lung cytokine expression. Interestingly, co-infected lungs had a lower expression of IFN-*γ* despite the *Trichuris*-driven Th1 response, and it was actually an IL-10-dominated response that appeared to limit antilarval schistosomula immunity (Bickle *et al*., 2008) and allowed progression of the parasite to the portal system with resulting increased egg burden and pathology in co-infected mice. Conversely, a chronic *T. muris* infection can be resolved by a *Schistosome* coinfection due to the *S. mansoni* egg-induced Th2 response (Curry *et al*., 1995). Additionally, *S. mansoni* and *T. muris* coinfected mice had significantly higher burden of adult *Schistosome* worms and eggs in the liver (Bickle *et al*., 2008) thus demonstrating that contrasting effects that the infections can have on one another.

## Trichuris and neoplasia

Cancer is a leading cause of death in high-income countries and incidences are increasing in low-income countries. There exists a strong link between inflammation and cancer with chronic infection and the long-term exposure to inflammatory stimuli heightening the risk of neoplastic change (Wang and Wang, 2007).

Chronic *T. muris* infection at day 80 p.i. in a wild-type mouse led to the development of neoplastic change that was similar to that seen in mice that had been treated with the carcinogen azoxymethane (Hayes *et al*., 2017). Intestinal crypt structure was altered alongside increased incidence of pre-adenomas which were more pronounced (in the case of aberrant crypt foci) in the infected mice as compared to the chemically treated mice. Even though *T. muris* infection can lead to increased epithelial proliferation and apoptosis in the intestine (Artis *et al*., 1999; Cliffe *et al*., 2007), both of which can lead to tumour formation (Evan and Vousden, 2001) these intestinal changes were only apparent in the caecum, the parasite niche, rather than throughout the small intestinal tract where neoplastic change was mostly observed (Hayes *et al*., 2017). Neoplastic change was seen in chronically infected animals even before the peak of parasite-specific cytokine responses was evident in the draining lymph node, although greater significant differences were seen as infection progressed. Infection generated a Th1-predominant response in these animals, however, this was not associated with a reduced neoplasia as might have been expected (Wang *et al*., 2015).

The APC^min/+^ tumour model in the mouse develops spontaneous adenomas throughout the GI tract (Moser *et al*., 1990). Chronic infection of APC^min/+^ mice with *T. muris* led to a significant increase in new tumour formation throughout the intestine and not just an increase in tumour size. Blockade of the CD25+ Treg response abrogated this heightened tumour formation demonstrating the role of the *T. muris*-induced Tregs in regulating the anti-tumour response in these animals (Hayes *et al*., 2017). Tregs have also been characterized within tumour microenvironments that can induce tumour-specific immune tolerance (Wang and Wang, 2007). Clonal expansion of tumour Tregs is thought to occur both locally and systemically and a high proportion of Tregs with the tumour micro-environment is correlative with poor prognosis in many cancer types suggestive of the suppressive role of Tregs on anti-tumour immunity (Mougiakakos, 2011; Fridman *et al*., 2012; Ahmadzadeh *et al*., 2019). Interestingly *T. suis* E/S proteins are capable of stimulating the secretion of IL-10 from macrophages though failed to induce CD25^+^Foxp3^+^ T cells unlike *T. muris* E/S which was able to do this (D'Elia *et al*., 2009; Leroux *et al*., 2018). Additionally, increased mucosal T cell activation production of IL-10, TGF-*β* and FoxP3 were found in the colon of an individual with ulcerative colitis who self-infected with *T. trichiura* (Dige *et al*., 2017). Tregs are known to play a role in both pathology and immunity early on following chronic *T. muris* infection as are TGF-*β* and IL-10 (D'Elia *et al*., 2009; Worthington *et al*., 2013; Sawant *et al*., 2014; Duque-Correa *et al*., 2019). It is noteworthy however, that low-dose chronic infection with *T. muris* is associated with a depression in Foxp3^+^CD4^+^T cells in the caecum and colon (Holm *et al*., 2015; Houlden *et al*., 2015). Taken together these data suggest that distinct populations of CD4^+^ T cells are involved in regulating tumours at sites away from the parasite niche.

IL-10 and TGF-*β* are not the only regulatory cytokines associated with a *T. muris* infection and cancer. IL-35 is an immune-suppressive cytokine which belongs to the IL-12 cytokine family and can also act to regulate Th1 immunity (Collison *et al*., 2007). Chronic *T. muris* infection can drive an inducible cell type (iT(R)35 cells) that exert regulatory effects *via* IL-35 and are Foxp3 independent (Collison *et al*., 2010). In a melanoma model of cancer, these *T. muris*-induced cells can be found within the tumour micro-environment (in the skin) and contributed to tumour progression by again regulating the ongoing anti-tumour responses (Collison *et al*., 2010). In addition, IL-31 is a Th2 T cell cytokine that can suppress type 2 immune responses (Dillon *et al*., 2004). IL-31 and IL31R play a regulatory role in *T. muris* infection with an induced production of this cytokine in the intestine following infection (Perrigoue *et al*., 2009). Additionally, infection of IL31R KO mice led to a heightened Th2 cytokine response and enhanced goblet cell hyperplasia with a resulting accelerated expulsion of worms. As this cytokine has also been implicated in cancer progression, it is likely that *T. muris* induced IL-31 production may also enhance tumour progression in a manner similar to IL-35 (He *et al*., 2020).

## Conclusion

*T. muris* is an intestinal dwelling nematode parasite that can have far-reaching consequences in the host (Fig. 1). Within the intestine itself, chronic *T. muris* in susceptible strains can have pathological consequences that show a degree of similarity to symptoms of IBD. Indeed, several genes upregulated during a chronic *T. muris* infection are also found to be upregulated in IBD patients. Paradoxically, *T. muris* infections can also help modulate IBD symptoms and pathologies due to the parasite-specific Treg response driven by infection. *T. muris* also drives microbiota changes in the host, beneficial to its survival, that have consequences for the host due to the impact that these changes can have on mucus constituents and intestinal permeability. Distal from the site of infection, *T. muris* infections can have an impact on immune responses to chemical sensitizers in the ear. In this case, a chronic *T. muris* driven IL-10 production preventing the egress of DCs from the ear. Chronic *T. muris* infection can also modulate immune responses in the lung to airway allergens which was also associated with an increased IL-10 response. *T. muris* infection can also influence immune responses in the brain and it has been demonstrated that an on-going *T. muris*-driven Th1 response will worsen the damage caused by experimental stroke, a process driven by an elevated and sustained RANTES production. Additionally, *T. muris* can have an effect on other brain inflammations with papers reporting changes in prion diseases and Alzheimer's progression. Although relatively little work has addressed the effects of *T. muris* on other parasite, viral and microbial infections, altered immunity to mycobacteria, pneumococcus, *N. brasiliensis, H. bakerii* and *S. mansoni* have been reported. Finally, effects of *T. muris* infection on cancer progression establish that the *T. muris*-driven Treg response plays an important role in inhibiting host immunity to adenoma progression in the intestine leading to development of more tumours. Additionally, two other regulatory cytokines, IL-35 and IL-31, induced by *T. muris* infection are able to modulate tumour immunity. In light of this, the importance of *T. muris* infections on other diseases and other body systems is profound and warrants further research and investigation, especially considering the widespread nature of this parasite in the human population.

## References

[ref1] Ahmadzadeh M, Pasetto A, Jia L, Deniger DC, Stevanović S, Robbins PF and Rosenberg SA (2019) Tumor-infiltrating human CD4(+) regulatory T cells display a distinct TCR repertoire and exhibit tumor and neoantigen reactivity. Science Immunology 4(31), eaao4310. doi: 10.1126/sciimmunol.aao4310 30635355 PMC6685542

[ref2] Aira N, Andersson AM, Singh SK, McKay DM and Blomgran R (2017) Species dependent impact of helminth-derived antigens on human macrophages infected with *Mycobacterium tuberculosis*: direct effect on the innate anti-mycobacterial response. PLoS Neglected Tropical Diseases 11, e0005390. 28192437 10.1371/journal.pntd.0005390 PMC5325601

[ref3] Ajendra J, Chenery AL, Parkinson JE, Chan BHK, Pearson S, Colombo SAP, Boon L, Grencis RK, Sutherland TE and Allen JE (2020) IL-17A both initiates, *via* IFN*γ* suppression, and limits the pulmonary type-2 immune response to nematode infection. Mucosal Immunology 13, 958–968. 32636457 10.1038/s41385-020-0318-2 PMC7567645

[ref4] Alcântara-Neves NM, Badaró SJ, dos Santos MC, Pontes-de-Carvalho L and Barreto ML (2010) The presence of serum anti-*Ascaris lumbricoides* IgE antibodies and of *Trichuris trichiura* infection are risk factors for wheezing and/or atopy in preschool-aged Brazilian children. Respiratory Researcher 11, 114. 10.1186/1465-9921-11-114 PMC2939601 20731833

[ref5] Andrews C, McLean MH and Durum SK (2016) Interleukin-27 as a novel therapy for inflammatory bowel disease: a critical review of the literature. Inflammatory Bowel Diseases 22, 2255–2264. 27243591 10.1097/MIB.0000000000000818 PMC4992429

[ref6] Arijs I, Li K, Toedter G, Quintens R, Van Lommel L, Van Steen K, Leemans P, De Hertogh G, Lemaire K, Ferrante M, Schnitzler F, Thorrez L, Ma K, Song XY, Marano C, Van Assche G, Vermeire S, Geboes K, Schuit F, Baribaud F and Rutgeerts P (2009) Mucosal gene signatures to predict response to infliximab in patients with ulcerative colitis. Gut 58, 1612–1619. 19700435 10.1136/gut.2009.178665

[ref7] Arijs I, Quintens R, Van Lommel L, Van Steen K, De Hertogh G, Lemaire K, Schraenen A, Perrier C, Van Assche G, Vermeire S, Geboes K, Schuit F and Rutgeerts P (2010) Predictive value of epithelial gene expression profiles for response to infliximab in Crohn's disease. Inflammatory Bowel Disease 16, 2090–2098. 10.1002/ibd.21301 20848504

[ref8] Artis D, Potten CS, Else KJ, Finkelman FD and Grencis RK (1999) *Trichuris muris*: host intestinal epithelial cell hyperproliferation during chronic infection is regulated by interferon-gamma. Experimental Parasitology 92, 144–153. 10366539 10.1006/expr.1999.4407

[ref9] Artis D, Villarino A, Silverman M, He W, Thornton EM, Mu S, Summer S, Covey TM, Huang E, Yoshida H, Koretzky G, Goldschmidt M, Wu GD, de Sauvage F, Miller HR, Saris CJ, Scott P and Hunter CA (2004) The IL-27 receptor (WSX-1) is an inhibitor of innate and adaptive elements of type 2 immunity. Journal of Immunology 173, 5626–5634. 10.4049/jimmunol.173.9.5626 15494513

[ref10] Awasthi A, Carrier Y, Peron JP, Bettelli E, Kamanaka M, Flavell RA, Kuchroo VK, Oukka M and Weiner HL (2007) A dominant function for interleukin 27 in generating interleukin 10-producing anti-inflammatory T cells. Nature Immunology 8, 1380–1389. 17994022 10.1038/ni1541

[ref11] Bancroft AJ, McKenzie AN and Grencis RK (1998) A critical role for IL-13 in resistance to intestinal nematode infection. Journal of Immunology 160, 3453–361. 9531306

[ref12] Bancroft AJ, Humphreys NE, Worthington JJ, Yoshida H and Grencis RK (2004) WSX-1: a key role in induction of chronic intestinal nematode infection. Journal of Immunology 172, 7635–7641. 10.4049/jimmunol.172.12.7635 15187144

[ref13] Bancroft AJ, Levy CW, Jowitt TA, Hayes KS, Thompson S, McKenzie EA, Ball MD, Dubaissi E, France AP, Bellina B, Sharpe C, Mironov A, Brown SL, Cook PC, MacDonald AS, Thornton DJ and Grencis RK (2019) The major secreted protein of the whipworm parasite tethers to matrix and inhibits interleukin-13 function. Nature Communications 10, 2344. 10.1038/s41467-019-09996-z PMC6538607 31138806

[ref14] Behnke JM, Ali NM and Jenkins SN (1984) Survival to patency of low level infections with *Trichuris muris* in mice concurrently infected with *Nematospiroides dubius*. Annals of Tropical Medicine and Parasitology 78, 509–517. 6524995 10.1080/00034983.1984.11811857

[ref15] Bickle QD, Solum J and Helmby H (2008) Chronic intestinal nematode infection exacerbates experimental *Schistosoma mansoni* infection. Infection and Immunity 76, 5802–5809. 18824532 10.1128/IAI.00827-08 PMC2583585

[ref16] Boros DL (1989) Immunopathology of *Schistosoma mansoni* infection. Clinical Microbiology Reviews 2, 250–269. 2504481 10.1128/cmr.2.3.250 PMC358119

[ref17] Bouchery T, Volpe B, Shah K, Lebon L, Filbey K, LeGros G and Harris N (2017) The study of host immune responses elicited by the model murine hookworms *Nippostrongylus brasiliensis* and *Heligmosomoides polygyrus*. Current Protocols in Mouse Biology 7, 236–286. 29261231 10.1002/cpmo.34

[ref18] Bourke CD, Mutapi F, Nausch N, Photiou DM, Poulsen LK, Kristensen B, Arnved J, Rønborg S, Roepstorff A, Thamsborg S, Kapel C, Melbye M and Bager P (2012) *Trichuris suis* ova therapy for allergic rhinitis does not affect allergen-specific cytokine responses despite a parasite-specific cytokine response. Clinical Experimental Allergy 42, 1582–1595. 23106658 10.1111/j.1365-2222.2012.04063.x

[ref19] Broadhurst MJ, Leung JM, Kashyap V, McCune JM, Mahadevan U, McKerrow JH and Loke P (2010) IL-22+ CD4+ T cells are associated with therapeutic *Trichuris trichiura* infection in an ulcerative colitis patient. Science Translational Medicine 2, 60ra88. 10.1126/scitranslmed.3001500 21123809

[ref20] Chen Z, Luo J, Li J, Kim G, Stewart A, Urban JF Jr., Huang Y, Chen S, Wu LG, Chesler A, Trinchieri G, Li W and Wu C (2021) Interleukin-33 promotes serotonin release from enterochromaffin cells for intestinal homeostasis. Immunity 54, 151–63.e6. 33220232 10.1016/j.immuni.2020.10.014 PMC7856083

[ref21] Chenery AL, Antignano F, Burrows K, Scheer S, Perona-Wright G and Zaph C (2016) Low-dose intestinal *Trichuris muris* infection alters the lung immune microenvironment and can suppress allergic airway inflammation. Infection and Immunity 84, 491–501. 26644379 10.1128/IAI.01240-15 PMC4730564

[ref22] Cliffe LJ and Grencis RK (2004) The *Trichuris muris* system: a paradigm of resistance and susceptibility to intestinal nematode infection. Advances in Parasitology 57, 255–307. 15504540 10.1016/S0065-308X(04)57004-5

[ref23] Cliffe LJ, Humphreys NE, Lane TE, Potten CS, Booth C and Grencis RK (2005) Accelerated intestinal epithelial cell turnover: a new mechanism of parasite expulsion. Science (New York, N.Y.) 308, 1463–1465. 10.1126/science.1108661 15933199

[ref24] Cliffe LJ, Potten CS, Booth CE and Grencis RK (2007) An increase in epithelial cell apoptosis is associated with chronic intestinal nematode infection. Infection and Immunity 75, 1556–1564. 17242061 10.1128/IAI.01375-06 PMC1865698

[ref25] Collison LW, Workman CJ, Kuo TT, Boyd K, Wang Y, Vignali KM, Cross R, Sehy D, Blumberg RS and Vignali DA (2007) The inhibitory cytokine IL-35 contributes to regulatory T-cell function. Nature 450, 566–569. 18033300 10.1038/nature06306

[ref26] Collison LW, Chaturvedi V, Henderson AL, Giacomin PR, Guy C, Bankoti J, Finkelstein D, Forbes K, Workman CJ, Brown SA, Rehg JE, Jones ML, Ni HT, Artis D, Turk MJ and Vignali DA (2010) IL-35-mediated induction of a potent regulatory T cell population. Nature Immunology 11, 1093–1101. 20953201 10.1038/ni.1952 PMC3008395

[ref27] Cooper ES, Bundy DA, MacDonald TT and Golden MH (1990) Growth suppression in the *Trichuris* dysentery syndrome. European Journal of Clinical Nutrition 44, 285–91. 2364918

[ref28] Cooper P, Walker AW, Reyes J, Chico M, Salter SJ, Vaca M and Parkhill J (2013) Patent human infections with the whipworm, *Trichuris trichiura*, are not associated with alterations in the faecal microbiota. PLoS One 8, e76573. 24124574 10.1371/journal.pone.0076573 PMC3790696

[ref29] Craig JM and Scott AL (2014) Helminths in the lungs. Parasite Immunology 36, 463–474. 25201409 10.1111/pim.12102

[ref30] Cumberbatch M, Dearman RJ, Griffiths CE and Kimber I (2000) Langerhans cell migration. Clinical and Experimental Dermatology 25, 413–418. 11012599 10.1046/j.1365-2230.2000.00678.x

[ref31] Curry AJ, Else KJ, Jones F, Bancroft A, Grencis RK and Dunne DW (1995) Evidence that cytokine-mediated immune interactions induced by *Schistosoma mansoni* alter disease outcome in mice concurrently infected with *Trichuris muris*. Journal of Experimental Medicine 181, 769–774. 10.1084/jem.181.2.769 PMC2191884 7836929

[ref32] D'Elia R, Behnke JM, Bradley JE and Else KJ (2009) Regulatory T cells: a role in the control of helminth-driven intestinal pathology and worm survival. Journal of Immunology 182, 2340–2348. 10.4049/jimmunol.0802767 PMC2649429 19201888

[ref33] Dénes A, Humphreys N, Lane TE, Grencis R and Rothwell N (2010) Chronic systemic infection exacerbates ischemic brain damage *via* a CCL5 (regulated on activation, normal T-cell expressed and secreted)-mediated proinflammatory response in mice. Journal of Neuroscience 30, 10086–10095. 20668193 10.1523/JNEUROSCI.1227-10.2010 PMC3044869

[ref34] de Waal Malefyt R, Figdor CG, Huijbens R, Mohan-Peterson S, Bennett B, Culpepper J, Dang W, Zurawski G and de Vries JE (1993) Effects of IL-13 on phenotype, cytokine production, and cytotoxic function of human monocytes. Comparison with IL-4 and modulation by IFN-gamma or IL-10. Journal of Immunology 151, 6370–6381. 7902377

[ref35] Dhungana H, Malm T, Denes A, Valonen P, Wojciechowski S, Magga J, Savchenko E, Humphreys N, Grencis R, Rothwell N and Koistinaho J (2013) Aging aggravates ischemic stroke-induced brain damage in mice with chronic peripheral infection. Aging Cell 12, 842–850. 23725345 10.1111/acel.12106

[ref36] Dige A, Rasmussen TK, Nejsum P, Hagemann-Madsen R, Williams AR, Agnholt J, Dahlerup JF and Hvas CL (2017) Mucosal and systemic immune modulation by *Trichuris trichiura* in a self-infected individual. Parasite Immunology 39(1), e12394. doi: 10.1111/pim.12394 27743501

[ref37] Dillon SR, Sprecher C, Hammond A, Bilsborough J, Rosenfeld-Franklin M, Presnell SR, Haugen HS, Maurer M, Harder B, Johnston J, Bort S, Mudri S, Kuijper JL, Bukowski T, Shea P, Dong DL, Dasovich M, Grant FJ, Lockwood L, Levin SD, LeCiel C, Waggie K, Day H, Topouzis S, Kramer J, Kuestner R, Chen Z, Foster D, Parrish-Novak J and Gross JA (2004) Interleukin 31, a cytokine produced by activated T cells, induces dermatitis in mice. Nature Immunology 5, 752–760. 15184896 10.1038/ni1084

[ref38] Doe C, Bafadhel M, Siddiqui S, Desai D, Mistry V, Rugman P, McCormick M, Woods J, May R, Sleeman MA, Anderson IK and Brightling CE (2010) Expression of the T helper 17-associated cytokines IL-17A and IL-17F in asthma and COPD. Chest 138, 1140–1147. 20538817 10.1378/chest.09-3058 PMC2972626

[ref39] Donaldson DS, Bradford BM, Else KJ and Mabbott NA (2020) Accelerated onset of CNS prion disease in mice co-infected with a gastrointestinal helminth pathogen during the preclinical phase. Scientific Reports 10, 4554. 32165661 10.1038/s41598-020-61483-4 PMC7067812

[ref40] Duque-Correa MA, Karp NA, McCarthy C, Forman S, Goulding D, Sankaranarayanan G, Jenkins TP, Reid AJ, Cambridge EL, Ballesteros Reviriego C, Müller W, Cantacessi C, Dougan G, Grencis RK and Berriman M (2019) Exclusive dependence of IL-10R*α* signalling on intestinal microbiota homeostasis and control of whipworm infection. PLoS Pathogens 15, e1007265. 30640950 10.1371/journal.ppat.1007265 PMC6347331

[ref41] Ebner F, Hepworth MR, Rausch S, Janek K, Niewienda A, Kühl A, Henklein P, Lucius R, Hamelmann E and Hartmann S (2014) Therapeutic potential of larval excretory/secretory proteins of the pig whipworm *Trichuris suis* in allergic disease. Allergy 69, 1489–1497. 25069662 10.1111/all.12496

[ref42] Else KJ and Grencis RK (1991) Cellular immune responses to the murine nematode parasite *Trichuris muris*. I. Differential cytokine production during acute or chronic infection. Immunology 72, 508–13. 1903765 PMC1384369

[ref43] Else KJ, Hültner L and Grencis RK (1992) Cellular immune responses to the murine nematode parasite *Trichuris muris*. II. Differential induction of TH-cell subsets in resistant versus susceptible mice. Immunology 75, 232–27. 1532377 PMC1384699

[ref44] Evan GI and Vousden KH (2001) Proliferation, cell cycle and apoptosis in cancer. Nature 411, 342–348. 11357141 10.1038/35077213

[ref45] Filbey KJ, Camberis M, Chandler J, Turner R, Kettle AJ, Eichenberger RM, Giacomin P and Le Gros G (2019) Intestinal helminth infection promotes IL-5- and CD4(+) T cell-dependent immunity in the lung against migrating parasites. Mucosal Immunology 12, 352–362. 30401814 10.1038/s41385-018-0102-8

[ref46] Fleming J, Hernandez G, Hartman L, Maksimovic J, Nace S, Lawler B, Risa T, Cook T, Agni R, Reichelderfer M, Luzzio C, Rolak L, Field A and Fabry Z (2019) Safety and efficacy of helminth treatment in relapsing-remitting multiple sclerosis: results of the HINT 2 clinical trial. Multiple Sclerosis 25, 81–91. 29064315 10.1177/1352458517736377 PMC5878983

[ref47] Foth BJ, Tsai IJ, Reid AJ, Bancroft AJ, Nichol S, Tracey A, Holroyd N, Cotton JA, Stanley EJ, Zarowiecki M, Liu JZ, Huckvale T, Cooper PJ, Grencis RK and Berriman M (2014) Whipworm genome and dual-species transcriptome analyses provide molecular insights into an intimate host-parasite interaction. Nature Genetics 46, 693–700. 24929830 10.1038/ng.3010 PMC5012510

[ref48] Fridman WH, Pagès F, Sautès-Fridman C and Galon J (2012) The immune contexture in human tumours: impact on clinical outcome. Nature Reviews Cancer 12, 298–306. 22419253 10.1038/nrc3245

[ref49] Fuss IJ, Neurath M, Boirivant M, Klein JS, de la Motte C, Strong SA, Fiocchi C and Strober W (1996) Disparate CD4+ lamina propria (LP) lymphokine secretion profiles in inflammatory bowel disease. Crohn's disease LP cells manifest increased secretion of IFN-gamma, whereas ulcerative colitis LP cells manifest increased secretion of IL-5. Journal of Immunology 157, 1261–1270. 8757634

[ref50] Fuss IJ, Heller F, Boirivant M, Leon F, Yoshida M, Fichtner-Feigl S, Yang Z, Exley M, Kitani A, Blumberg RS, Mannon P and Strober W (2004) Nonclassical CD1d-restricted NK T cells that produce IL-13 characterize an atypical Th2 response in ulcerative colitis. Journal of Clinical Investigation 113, 1490–1497. 10.1172/JCI19836 PMC406524 15146247

[ref51] Glover M, Colombo SAP, Thornton DJ and Grencis RK (2019) Trickle infection and immunity to *Trichuris muris*. PLoS Pathogens 15, e1007926. 31730667 10.1371/journal.ppat.1007926 PMC6881069

[ref52] Gomaa EZ (2020) Human gut microbiota/microbiome in health and diseases: a review. Antonie Van Leeuwenhoek 113, 2019–2040. 33136284 10.1007/s10482-020-01474-7

[ref53] Gonçales JP, Nobrega CGO, Nascimento WRC, Lorena VMB, Peixoto DM, Costa VMA, Barbosa CS, Solé D, Sarinho ESC and Souza VMO (2020) Cytokine production in allergic and *Trichuris trichiura*-infected children from an urban region of the Brazilian northeast. Parasitology International 74, 101918. 31004803 10.1016/j.parint.2019.04.015

[ref54] Grencis RK, Humphreys NE and Bancroft AJ (2014) Immunity to gastrointestinal nematodes: mechanisms and myths. Immunology Reviews 260, 183–205. 10.1111/imr.12188 PMC4141702 24942690

[ref55] Guan Q (2019) A comprehensive review and update on the pathogenesis of inflammatory bowel disease. Journal of Immunology Research 2019, 7247238. 31886308 10.1155/2019/7247238 PMC6914932

[ref56] Gurczynski SJ and Moore BB (2018) IL-17 in the lung: the good, the bad, and the ugly. American Journal of Physiology -Lung Cellular and Molecular Physiology 314, L6–l16. 28860146 10.1152/ajplung.00344.2017 PMC6048455

[ref57] Hansen CS, Hasseldam H, Bacher IH, Thamsborg SM, Johansen FF and Kringel H (2017) *Trichuris suis* secrete products that reduce disease severity in a multiple sclerosis model. Acta Parasitologica 62, 22–28. 28030334 10.1515/ap-2017-0002

[ref58] Hanson ML, Hixon JA, Li W, Felber BK, Anver MR, Stewart CA, Janelsins BM, Datta SK, Shen W, McLean MH and Durum SK (2014) Oral delivery of IL-27 recombinant bacteria attenuates immune colitis in mice. Gastroenterology 146, 210–21.e13. 24120477 10.1053/j.gastro.2013.09.060 PMC3920828

[ref59] Hasnain SZ, Wang H, Ghia JE, Haq N, Deng Y, Velcich A, Grencis RK, Thornton DJ and Khan WI (2010) Mucin gene deficiency in mice impairs host resistance to an enteric parasitic infection. Gastroenterology 138, 1763–1771. 20138044 10.1053/j.gastro.2010.01.045 PMC3466424

[ref60] Hasnain SZ, Thornton DJ and Grencis RK (2011) Changes in the mucosal barrier during acute and chronic *Trichuris muris* infection. Parasite Immunology 33, 45–55. 21155842 10.1111/j.1365-3024.2010.01258.x PMC3020324

[ref61] Hasnain SZ, McGuckin MA, Grencis RK and Thornton DJ (2012) Serine protease(s) secreted by the nematode *Trichuris muris* degrade the mucus barrier. PLoS Neglected Tropical Diseases 6, e1856. 23071854 10.1371/journal.pntd.0001856 PMC3469553

[ref62] Hayes KS, Cliffe LJ, Bancroft AJ, Forman SP, Thompson S, Booth C and Grencis RK (2017) Chronic *Trichuris muris* infection causes neoplastic change in the intestine and exacerbates tumour formation in APC min/+ mice. PLoS Neglected Tropical Diseases 11, e0005708. 28650985 10.1371/journal.pntd.0005708 PMC5501682

[ref63] He Y, Zhang X, Pan W, Tai F, Liang L and Shi J (2020) Interleukin-31 receptor *α* is required for basal-like breast cancer progression. Frontiers in Oncology 10, 816. 32528891 10.3389/fonc.2020.00816 PMC7266966

[ref64] Hiemstra IH, Klaver EJ, Vrijland K, Kringel H, Andreasen A, Bouma G, Kraal G, van Die I and den Haan JM (2014) Excreted/secreted *Trichuris suis* products reduce barrier function and suppress inflammatory cytokine production of intestinal epithelial cells. Molecular Immunology 60, 1–7. 24705296 10.1016/j.molimm.2014.03.003

[ref65] Holm JB, Sorobetea D, Kiilerich P, Ramayo-Caldas Y, Estellé J, Ma T, Madsen L, Kristiansen K and Svensson-Frej M (2015) Chronic *Trichuris muris* infection decreases diversity of the intestinal microbiota and concomitantly increases the abundance of lactobacilli. PLoS One 10, e0125495. 25942314 10.1371/journal.pone.0125495 PMC4420551

[ref66] Hotez PJ, Alvarado M, Basáñez MG, Bolliger I, Bourne R, Boussinesq M, Brooker SJ, Brown AS, Buckle G, Budke CM, Carabin H, Coffeng LE, Fèvre EM, Fürst T, Halasa YA, Jasrasaria R, Johns NE, Keiser J, King CH, Lozano R, Murdoch ME, O'Hanlon S, Pion SD, Pullan RL, Ramaiah KD, Roberts T, Shepard DS, Smith JL, Stolk WA, Undurraga EA, Utzinger J, Wang M, Murray CJ and Naghavi M (2014) The global burden of disease study 2010: interpretation and implications for the neglected tropical diseases. PLoS Neglected Tropical Diseases 8, e2865. 25058013 10.1371/journal.pntd.0002865 PMC4109880

[ref67] Houlden A, Hayes KS, Bancroft AJ, Worthington JJ, Wang P, Grencis RK and Roberts IS (2015) Chronic *Trichuris muris* infection in C57BL/6 mice causes significant changes in host microbiota and metabolome: effects reversed by pathogen clearance. PLoS One 10, e0125945. 25938477 10.1371/journal.pone.0125945 PMC4418675

[ref68] Karmele EP, Pasricha TS, Ramalingam TR, Thompson RW, Gieseck RL III, Knilans KJ, Hegen M, Farmer M, Jin F, Kleinman A, Hinds DA, Almeida Pereira T, de Queiroz Prado R, Bing N, Tchistiakova L, Kasaian MT, Wynn TA and Vannella KM (2019) Anti-IL-13R*α*2 therapy promotes recovery in a murine model of inflammatory bowel disease. Mucosal Immunology 12, 1174–1186. 31308480 10.1038/s41385-019-0189-6 PMC6717533

[ref69] Khan WI, Richard M, Akiho H, Blennerhasset PA, Humphreys NE, Grencis RK, Van Snick J and Collins SM (2003) Modulation of intestinal muscle contraction by interleukin-9 (IL-9) or IL-9 neutralization: correlation with worm expulsion in murine nematode infections. Infection and Immunity 71, 2430–2438. 12704113 10.1128/IAI.71.5.2430-2438.2003 PMC153239

[ref70] Kopper JJ, Patterson JS and Mansfield LS (2015) Metronidazole-but not IL-10 or prednisolone-rescues *Trichuris muris* infected C57BL/6 IL-10 deficient mice from severe disease. Veterinary Parasitology 212, 239–252. 26277566 10.1016/j.vetpar.2015.07.038

[ref71] Kuijk LM, Klaver EJ, Kooij G, van der Pol SM, Heijnen P, Bruijns SC, Kringel H, Pinelli E, Kraal G, de Vries HE, Dijkstra CD, Bouma G and van Die I (2012) Soluble helminth products suppress clinical signs in murine experimental autoimmune encephalomyelitis and differentially modulate human dendritic cell activation. Molecular Immunology 51, 210–218. 22482518 10.1016/j.molimm.2012.03.020

[ref72] Law AE, Shears RK, Lopez Rodas AA, Grencis RK, Cooper PJ, Neill DR and Kadioglu A (2021) Intestinal helminth co-infection is an unrecognised risk factor for increased pneumococcal carriage density and invasive disease. Scientific Reports 11, 6984. 33772094 10.1038/s41598-021-86508-4 PMC7997997

[ref73] Leroux LP, Nasr M, Valanparambil R, Tam M, Rosa BA, Siciliani E, Hill DE, Zarlenga DS, Jaramillo M, Weinstock JV, Geary TG, Stevenson MM, Urban JF Jr., Mitreva M and Jardim A (2018) Analysis of the *Trichuris suis* excretory/secretory proteins as a function of life cycle stage and their immunomodulatory properties. Scientific Reports 8, 15921. 30374177 10.1038/s41598-018-34174-4 PMC6206011

[ref74] Levison SE, McLaughlin JT, Zeef LA, Fisher P, Grencis RK and Pennock JL (2010) Colonic transcriptional profiling in resistance and susceptibility to trichuriasis: phenotyping a chronic colitis and lessons for iatrogenic helminthosis. Inflammatory Bowel Diseases 16, 2065–2079. 20687192 10.1002/ibd.21326

[ref75] Levison SE, Fisher P, Hankinson J, Zeef L, Eyre S, Ollier WE, McLaughlin JT, Brass A, Grencis RK and Pennock JL (2013) Genetic analysis of the *Trichuris muris*-induced model of colitis reveals QTL overlap and a novel gene cluster for establishing colonic inflammation. BMC Genomics 14, 127. 23442222 10.1186/1471-2164-14-127 PMC3621453

[ref76] Li MO and Flavell RA (2008) TGF-beta: a master of all T cell trades. Cell 134, 392–404. 18692464 10.1016/j.cell.2008.07.025 PMC3677783

[ref77] Li CS, Zhang Q, Lee KJ, Cho SW, Lee KM, Hahm KB, Choi SC, Yun KJ, Chung HT and Chae SC (2009) Interleukin-27 polymorphisms are associated with inflammatory bowel diseases in a Korean population. Journal of Gastroenterology and Hepatology 24, 1692–1696. 19686419 10.1111/j.1440-1746.2009.05901.x

[ref78] Li RW, Wu S, Li W, Navarro K, Couch RD, Hill D and Urban JF Jr. (2012) Alterations in the porcine colon microbiota induced by the gastrointestinal nematode *Trichuris suis*. Infection and Immunity 80, 2150–2157. 22493085 10.1128/IAI.00141-12 PMC3370577

[ref79] Linden SK, Sutton P, Karlsson NG, Korolik V and McGuckin MA (2008) Mucins in the mucosal barrier to infection. Mucosal Immunology 1, 183–197. 19079178 10.1038/mi.2008.5 PMC7100821

[ref80] MacDonald TT, Spencer J, Murch SH, Choy MY, Venugopal S, Bundy DA and Cooper ES (1994) Immunoepidemiology of intestinal helminthic infections. 3. Mucosal macrophages and cytokine production in the colon of children with *Trichuris trichiura* dysentery. Transactions of the Royal Society of Tropical Medicine and Hygiene 88, 265–268. 7974659 10.1016/0035-9203(94)90072-8

[ref81] Mankertz J and Schulzke JD (2007) Altered permeability in inflammatory bowel disease: pathophysiology and clinical implications. Current Opinion in Gastroenterology 23, 379–383. 17545772 10.1097/MOG.0b013e32816aa392

[ref82] Mentink-Kane MM and Wynn TA (2004) Opposing roles for IL-13 and IL-13 receptor alpha 2 in health and disease. Immunological Reviews 202, 191–202. 15546394 10.1111/j.0105-2896.2004.00210.x

[ref83] Metcalfe DD, Baram D and Mekori YA (1997) Mast cells. Physiological Reviews 77, 1033–1079. 9354811 10.1152/physrev.1997.77.4.1033

[ref84] Minty A, Chalon P, Derocq JM, Dumont X, Guillemot JC, Kaghad M, Labit C, Leplatois P, Liauzun P, Miloux B, Minty C, Casellas P, Loison G, Lupker J, Shire D, Ferrara P and Caput D (1993) Interleukin-13 is a new human lymphokine regulating inflammatory and immune responses. Nature 362, 248–250. 8096327 10.1038/362248a0

[ref85] Montacute R, Foley K, Forman R, Else KJ, Cruickshank SM and Allan SM (2017) Enhanced susceptibility of triple transgenic Alzheimer's disease (3xTg-AD) mice to acute infection. Journal of Neuroinflammation 14, 50. 28284226 10.1186/s12974-017-0826-5 PMC5346250

[ref86] Moser AR, Pitot HC and Dove WF (1990) A dominant mutation that predisposes to multiple intestinal neoplasia in the mouse. Science (New York, N.Y.) 247, 322–324. 10.1126/science.2296722 2296722

[ref87] Mougiakakos D (2011) Regulatory T cells in colorectal cancer: from biology to prognostic relevance. Cancers (Basel) 3, 1708–1731. 24212779 10.3390/cancers3021708 PMC3757386

[ref88] Nel HJ, du Plessis N, Kleynhans L, Loxton AG, van Helden PD and Walzl G (2014) *Mycobacterium bovis* BCG infection severely delays *Trichuris muris* expulsion and co-infection suppresses immune responsiveness to both pathogens. BMC Microbiology 14, 9. 24433309 10.1186/1471-2180-14-9 PMC3898725

[ref89] Nemeth ZH, Bogdanovski DA, Barratt-Stopper P, Paglinco SR, Antonioli L and Rolandelli RH (2017) Crohn's disease and ulcerative colitis show unique cytokine profiles. Cureus 9, e1177. 28533995 10.7759/cureus.1177 PMC5438231

[ref90] Parthasarathy G and Mansfield LS (2005) *Trichuris suis* excretory secretory products (ESP) elicit interleukin-6 (IL-6) and IL-10 secretion from intestinal epithelial cells (IPEC-1). Veterinary Parasitology 131, 317–324. 15978725 10.1016/j.vetpar.2005.03.043

[ref91] Perrigoue JG, Zaph C, Guild K, Du Y and Artis D (2009) IL-31-IL-31R interactions limit the magnitude of Th2 cytokine-dependent immunity and inflammation following intestinal helminth infection. Journal of Immunology 182, 6088–6094. 10.4049/jimmunol.0802459 PMC2828776 19414760

[ref92] Pflanz S, Timans JC, Cheung J, Rosales R, Kanzler H, Gilbert J, Hibbert L, Churakova T, Travis M, Vaisberg E, Blumenschein WM, Mattson JD, Wagner JL, To W, Zurawski S, McClanahan TK, Gorman DM, Bazan JF, de Waal Malefyt R, Rennick D and Kastelein RA (2002) IL-27, a heterodimeric cytokine composed of EBI3 and p28 protein, induces proliferation of naive CD4+ T cells. Immunity 16, 779–790. 12121660 10.1016/s1074-7613(02)00324-2

[ref93] Phillips RS and Wakelin D (1974) Suppression of immunity in mice in the nematode *Trichuris muris* by concurrent infection with rodent piroplasms. Transactions of the Royal Society of Tropical Medicine and Hygiene 68, 276. 4423755

[ref94] Phillips RS, Selby GR and Wakelin D (1974) The effect of *Plasmodium berghei* and *Trypanosoma brucei* infections on the immune expulsion of the nematode *Trichuris muris* from mice. International Journal of Parasitology 4, 409–415. 4615070 10.1016/0020-7519(74)90050-2

[ref95] Querfurth HW and LaFerla FM (2010) Alzheimer's disease. New England Journal of Medicine 362, 329–344. 10.1056/NEJMra0909142 20107219

[ref96] Ramanan D, Tang MS, Bowcutt R, Loke P and Cadwell K (2014) Bacterial sensor Nod2 prevents inflammation of the small intestine by restricting the expansion of the commensal *Bacteroides vulgatus*. Immunity 41, 311–324. 25088769 10.1016/j.immuni.2014.06.015 PMC4238935

[ref97] Ramanan D, Bowcutt R, Lee SC, Tang MS, Kurtz ZD, Ding Y, Honda K, Gause WC, Blaser MJ, Bonneau RA, Lim YA, Loke P and Cadwell K (2016) Helminth infection promotes colonization resistance *via* type 2 immunity. Science (New York, N.Y.) 352, 608–612. 10.1126/science.aaf3229 PMC4905769 27080105

[ref98] Rodrigues LC, Newcombe PJ, Cunha SS, Alcantara-Neves NM, Genser B, Cruz AA, Simoes SM, Fiaccone R, Amorim L, Cooper PJ and Barreto ML (2008) Early infection with *Trichuris trichiura* and allergen skin test reactivity in later childhood. Clinical and Experimental Allergy 38, 1769–1777. 18547322 10.1111/j.1365-2222.2008.03027.x

[ref99] Sawant DV, Gravano DM, Vogel P, Giacomin P, Artis D and Vignali DA (2014) Regulatory T cells limit induction of protective immunity and promote immune pathology following intestinal helminth infection. Journal of Immunology 192, 2904–2912. 10.4049/jimmunol.1202502 PMC3955731 24532574

[ref100] Schachter J, Alvarinho de Oliveira D, da Silva CM, de Barros Alencar ACM, Duarte M, da Silva MMP, Ignácio ACPR and Lopes-Torres EJ (2020) Whipworm infection promotes bacterial invasion, intestinal microbiota imbalance, and cellular immunomodulation. Infection and Immunology 88(3), e00642-19. doi: 10.1128/iai.00642-19 PMC7035941 31843966

[ref101] Schölmerich J, Fellermann K, Seibold FW, Rogler G, Langhorst J, Howaldt S, Novacek G, Petersen AM, Bachmann O, Matthes H, Hesselbarth N, Teich N, Wehkamp J, Klaus J, Ott C, Dilger K, Greinwald R and Mueller R (2017) A randomised, double-blind, placebo-controlled trial of *Trichuris suis* ova in active Crohn's disease. Journal of Crohn's & Colitis 11, 390–399. 10.1093/ecco-jcc/jjw184 PMC5881737 27707789

[ref102] Schopf LR, Hoffmann KF, Cheever AW, Urban JF Jr. and Wynn TA (2002) IL-10 is critical for host resistance and survival during gastrointestinal helminth infection. Journal of Immunology 168, 2383–2392. 10.4049/jimmunol.168.5.2383 11859129

[ref103] Schulzke JD, Bojarski C, Zeissig S, Heller F, Gitter AH and Fromm M (2006) Disrupted barrier function through epithelial cell apoptosis. Annals of the New York Academy of Sciences 1072, 288–299. 17057208 10.1196/annals.1326.027

[ref104] Sorobetea D, Holm JB, Henningsson H, Kristiansen K and Svensson-Frej M (2017) Acute infection with the intestinal parasite *Trichuris muris* has long-term consequences on mucosal mast cell homeostasis and epithelial integrity. European Journal of Immunology 47, 257–268. 27891580 10.1002/eji.201646738

[ref105] Strober W, Fuss I and Mannon P (2007) The fundamental basis of inflammatory bowel disease. Journal of Clinical Investigation 117, 514–521. 10.1172/JCI30587 PMC1804356 17332878

[ref106] Summers RW, Elliott DE, Urban JF Jr., Thompson RA and Weinstock JV (2005 *a*) *Trichuris suis* therapy for active ulcerative colitis: a randomized controlled trial. Gastroenterology 128, 825–832. 15825065 10.1053/j.gastro.2005.01.005

[ref107] Summers RW, Elliott DE, Urban JF Jr., Thompson R and Weinstock JV (2005 *b*) *Trichuris suis* therapy in Crohn's disease. Gut 54, 87–90. 15591509 10.1136/gut.2004.041749 PMC1774382

[ref108] te Velde AA, de Kort F, Sterrenburg E, Pronk I, ten Kate FJ, Hommes DW and van Deventer SJ (2007) Comparative analysis of colonic gene expression of three experimental colitis models mimicking inflammatory bowel disease. Inflammatory Bowel Diseases 13, 325–330. 17206675 10.1002/ibd.20079

[ref109] Villarino AV, Artis D, Bezbradica JS, Miller O, Saris CJ, Joyce S and Hunter CA (2008) IL-27R deficiency delays the onset of colitis and protects from helminth-induced pathology in a model of chronic IBD. International Immunology 20, 739–752. 18375937 10.1093/intimm/dxn032

[ref110] Voldsgaard A, Bager P, Garde E, Åkeson P, Leffers AM, Madsen CG, Kapel C, Roepstorff A, Thamsborg SM, Melbye M, Siebner H, Søndergaard HB, Sellebjerg F and Sørensen PS (2015) *Trichuris suis* ova therapy in relapsing multiple sclerosis is safe but without signals of beneficial effect. Multiple Sclerosis 21, 1723–1729. 25698173 10.1177/1352458514568173

[ref111] Wang HY and Wang RF (2007) Regulatory T cells and cancer. Current Opinion in Immunology 19, 217–223. 17306521 10.1016/j.coi.2007.02.004

[ref112] Wang Z, Wang L, Fan R, Zhou J and Zhong J (2014) Association of IL-27 gene three polymorphisms with Crohn's disease susceptibility in a Chinese Han population. International Journal of Clinical and Experimental Pathology 7, 8952–897. 25674271 PMC4313976

[ref113] Wang L, Wang Y, Song Z, Chu J and Qu X (2015) Deficiency of interferon-gamma or its receptor promotes colorectal cancer development. Journal of Interferon & Cytokine Research 35, 273–280. 25383957 10.1089/jir.2014.0132

[ref114] White EC, Houlden A, Bancroft AJ, Hayes KS, Goldrick M, Grencis RK and Roberts IS (2018) Manipulation of host and parasite microbiotas: survival strategies during chronic nematode infection. Science Advances 4, eaap7399. 29546242 10.1126/sciadv.aap7399 PMC5851687

[ref115] WHO (2020) Soil-transmitted Helminth Infections, Fact Sheet. Geneva, Switzerland: World Health Organization.

[ref116] Wilson MS, Ramalingam TR, Rivollier A, Shenderov K, Mentink-Kane MM, Madala SK, Cheever AW, Artis D, Kelsall BL and Wynn TA (2011) Colitis and intestinal inflammation in IL10-/- mice results from IL-13R*α*2-mediated attenuation of IL-13 activity. Gastroenterology 140, 254–264. 20951137 10.1053/j.gastro.2010.09.047 PMC3006653

[ref117] Worthington JJ, Klementowicz JE, Rahman S, Czajkowska BI, Smedley C, Waldmann H, Sparwasser T, Grencis RK and Travis MA (2013) Loss of the TGF*β*-activating integrin *α*v*β*8 on dendritic cells protects mice from chronic intestinal parasitic infection *via* control of type 2 immunity. PLoS Pathogens 9, e1003675. 24098124 10.1371/journal.ppat.1003675 PMC3789784

[ref118] Wynn TA (2015) Type 2 cytokines: mechanisms and therapeutic strategies. Nature Reviews Immunology 15, 271–282. 10.1038/nri3831 25882242

[ref119] Yordanova IA, Ebner F, Schulz AR, Steinfelder S, Rosche B, Bolze A, Paul F, Mei HE and Hartmann S (2021) The worm-specific immune response in multiple sclerosis patients receiving controlled *Trichuris suis* ova immunotherapy. Life (Basel) 11(2), 101. doi: 10.3390/life11020101 33572978 PMC7912101

[ref120] Yoshida H and Hunter CA (2015) The immunobiology of interleukin-27. Annual Review of Immunology 33, 417–443. 10.1146/annurev-immunol-032414-112134 25861977

